# Comparative Analysis of Mutant Huntingtin Binding Partners in Yeast Species

**DOI:** 10.1038/s41598-018-27900-5

**Published:** 2018-06-22

**Authors:** Yanding Zhao, Ashley A. Zurawel, Nicole P. Jenkins, Martin L. Duennwald, Chao Cheng, Arminja N. Kettenbach, Surachai Supattapone

**Affiliations:** 1 Departments of Molecular and Systems Biology, Geisel School of Medicine at Dartmouth College, Hanover, New Hampshire 03755 United States; 2 Biochemistry and Cell Biology, Geisel School of Medicine at Dartmouth College, Hanover, New Hampshire 03755 United States; 3 Department of Pathology, Schulich School of Medicine and Dentistry, University of Western Ontario, London, Ontario, N6A 5C1 Canada; 4 Biomedical Data Sciences, Geisel School of Medicine at Dartmouth College, Hanover, New Hampshire 03755 United States; 5 Medicine, Geisel School of Medicine at Dartmouth College, Hanover, New Hampshire 03755 United States

## Abstract

Huntington’s disease is caused by the pathological expansion of a polyglutamine (polyQ) stretch in *Huntingtin* (*Htt*), but the molecular mechanisms by which polyQ expansion in Htt causes toxicity in selective neuronal populations remain poorly understood. Interestingly, heterologous expression of expanded polyQ Htt is toxic in *Saccharomyces cerevisiae* cells, but has no effect in *Schizosaccharomyces pombe*, a related yeast species possessing very few endogenous polyQ or Q/N-rich proteins. Here, we used a comprehensive and unbiased mass spectrometric approach to identify proteins that bind *Htt* in a length-dependent manner in both species. Analysis of the expanded polyQ-associated proteins reveals marked enrichment of proteins that are localized to and play functional roles in nucleoli and mitochondria in *S. cerevisiae*, but not in *S. pombe*. Moreover, expanded polyQ Htt appears to interact preferentially with endogenous polyQ and Q/N-rich proteins, which are rare in *S. pombe*, as well as proteins containing coiled-coil motifs in *S. cerevisiae*. Taken together, these results suggest that polyQ expansion of Htt may cause cellular toxicity in *S. cerevisiae* by sequestering endogenous polyQ and Q/N-rich proteins, particularly within nucleoli and mitochondria.

## Introduction

Proteins containing polyglutamine (polyQ) stretches (defined as sequences of >10 consecutive glutamine residues) are expressed in all known eukaryotic species^1^. PolyQ proteins are believed to facilitate protein-protein interactions^2–4^, and participate in a wide range of biological functions, including cell cycle regulation^5^, transcriptional regulation, and chromatin maintenance^1^. The distribution of polyQ proteins varies greatly between different species; for instance, ~5% and ~11% of all proteins in *Drosophila melanogaster* and *Dictyostelium discoideum*, respectively, contain polyQ stretches, whereas only ~0.07% of all proteins in *Schizosaccharomyces pombe* contain polyQ stretches^1^.

In humans, a group of related monogenic neurodegenerative diseases are caused by mutations of specific polyQ proteins, which cause expansion of the polyQ stretches within those proteins beyond a threshold length^6^. For instance, Huntington’s disease is caused by expansion of the *Huntingtin* (*Htt*) polyQ stretch beyond 35 residues^7^. Mutant Htt accumulates within intra-nuclear inclusions, especially in medial spiny neurons of the striatum, and eventually causes neuronal dysfunction and death^8,9^. Toxicity from polyQ expansion appears to contribute significantly to the disease^10^, and polyQ-initiated neurodegeneration can be modelled by transgenic expression of expanded polyQ Htt in a variety of model organisms, including mice^11^, fruit flies^12^, and nematodes^13–15^.

Interestingly, expression of the *Htt* polyQ stretch also causes cellular toxicity in the budding yeast *Saccharomyces cerevisiae* in a polyQ length-dependent manner^16–18^, and there are many similarities in the pattern of toxicity induced by mutant Htt between neurons and *S. cerevisiae*^18^. In particular, polyQ toxicity in both cell types is characterized by: (1) interactions between mutant Htt with other polyQ and Q/N-rich (prion-like) proteins^19–29^; (2) defects in endoplasmic reticulum (ER) protein quality control^30,31^; (3) cytoskeletal changes^32^; (4) transcriptional dysregulation^33–35^; (5) mitochondrial dysfunction^31,32,36,37^; and (6) apoptosis^32^. Moreover, genetic screens in *S. cerevisiae* have identified modifiers for each of these processes, supporting their functional involvement in the cell death pathway^38–42^.

It has been hypothesized that mutant Htt initiates cell death by sequestering other proteins into aggregates, thereby making them unavailable to perform their normal regulatory or enzymatic functions^3,43^. Consistent with this hypothesis, there appears to be a general correlation between the presence of Htt aggregates and cell death in a variety of model organisms, including *S. cerevisiae*^19,24,44^ and *Dictyostelium discoideum*, in which a strong chaperone network prevents aggregate formation^45^. However, we recently reported that the fission yeast, *Schizosaccharomyces pombe*, a species that notably contains very few endogenous polyQ and Q/N-rich proteins, provides an exception to this correlation^46^. Although expression of 103Q-Htt in *S. pombe* produces intracellular aggregates, no cytotoxicity or growth defects are observed.

Here, we sought to perform a comprehensive and unbiased mass spectrometry/bioinformatic analysis of the endogenous proteins in both *S. cerevisiae* and *S. pombe* that selectively bind to Htt aggregates in a polyQ length-dependent manner. This quantitative analysis provides a unique opportunity to systematically study the effect of polyQ expansion on protein-protein interactions in two distantly-related yeast species with different levels of endogenous polyQ and Q/N-rich proteins and different toxicity phenotypes.

## Materials and Methods

### Yeast Strains and Methods

*S. cerevisiae* strains [P3nmt1-FLAG-HTT(25Q)- green fluorescent protein (GFP)::leu1 + leu1-32 h- and P3nmt1-FLAG-HTT(103Q)-GFP::leu1 + leu1-32 h] [PIN + ] were supplied by M.D. (University of Western Ontario, Canada)^25^. The GAL1 inducible promoter controls *Htt* expression in these strains. Growth and induction for expression was performed by growing strains in selective media using a 1% glucose/1% galactose carbon source (Complete supplement mixture (CSM)-his: MP Biomedicals, Santa Ana, CA; Yeast Nitrogen Base: US Biologicals Salem, MA; Sigma Aldrich, St. Louis, MO; Galactose: Sigma Aldrich, St. Louis, MO; Glucose: Thermo Fisher Scientific, Waltham, MA) to mid-log phase, and then pelleting and washing the cells three times with H_2_0 before re-suspending in selective media with a 2% galactose/0.2% glucose carbon source.

*S. pombe* strains [MATα PGAL1-FLAG-HTT(25Q)-CFP::his3 + can1-100 ade2-1 his3-11, 15 trp1-1 ura3-1 leu23,112 and MATα PGAL1-FLAG-HTT(103Q)-CFP::his3 + can1-100 ade2-1 his3-11, 15 trp1-1 ura3-1 leu23,112] were generated, as previously described^46^. Briefly, *S. pombe* strains and media were made using standard methods^47^, and transformed into JM837 (leu1-32 h-). The growth and induction of strains was performed in selective or Edinburgh minimal media (EMM: MP Biomedicals, Santa Ana, CA). containing 15 μM thiamine (Sigma Aldrich, St. Louis, MO) to mid-log phase, followed by pelleting and washing the cells three times with H_2_0 before resuspending them in selective or minimal media without thiamine.

### Immunoprecipitation Using Anti-FLAG Beads

Immunoprecipitation reactions were done in biological triplicates for each strain: *S. cerevisiae* Htt-25Q, *S. cerevisiae* Htt-97Q, *S. pombe* Htt-25Q, and *S. pombe* Htt-103Q, as previously described^4^ by growing yeast cells to mid-log phase and inducing protein expression. Cells were then grown to mid-log phase for 16 hrs and cells were collected and pelleted. Pellets were washed twice in buffer [20 mM 4-(2-hydroxyethyl)-1-piperazineethanesulfonic acid (HEPES) (Sigma Aldrich) pH 7.4, 1 mM ethylenediaminetetraacetic acid (EDTA) (Sigma Aldrich), 300 mM NaCl (Thermo Fisher Scientific), 0.2% Triton (Sigma) with EDTA-free protease inhibitors (Roche, Indianapolis, IN)] and transferred to screw-cap tubes. Half of the volume of glass beads were added and cells were lysed using a Mini-bead-beater 16 (Biospec, Bartlesville, OK) at 4 °C twice in 1-min bursts, keeping tubes chilled on ice in-between. Cell lysate was then transferred to a new microfuge tube by puncturing a hole in the bottom of the Eppendorf using a 25-G needle (BD Biosciences, San Jose, CA) and briefly spinning at 5,000 × *g*. Cells were then centrifuged for 5 minutes at 16,000 × *g* at 4 °C to pellet the cell debris. Supernatant was then taken and incubated with 25 μL (≥0.6 mg/mL binding capacity, resin in 50% suspension) anti-FLAG M2 Magnetic Beads (Sigma Aldrich, St. Louis, MO) for each 1.5 mL of cell lysate (equivalent to no more than 125 optical density at 600 nm (OD) of cells) by end-over-end rotation for 1 hr at 4 °C. Beads were washed thoroughly using 1.5 mL of the lysis buffer in four sequential washes, using a magnetic bead separator to take off the eluate in between steps. For large-scale experiments, at least 500 OD of cells were used for each sample; for small-scale experiments, 25 OD of cells were harvested. Samples were eluted by boiling the magnetic beads in 60 μL of modified sodium dodecyl sulfate polyacrylamide gel electrophoresis (SDS-PAGE) buffer [0.2 M Tris pH 6.8 (Invitrogen, Carlsbad, CA), 8% SDS (Sigma), 20% beta-mercaptoethanol (Omnipur, Billerica MA)] for 15 min.

Prior to mass spectrometry, aliquots of immunoprecipitated samples were analysed using SDS-PAGE gel electrophoresis using a 12% polyacrylamide gel followed by Coomassie staining (Thermo Fisher Scientific) or western blotting, as previously described^48^ using a monoclonal anti-FLAG M2 antibody (Sigma Aldrich) for primary detection.

### Mass Spectrometry

The remainder of each immunoprecipitated sample was TCA precipitated and digested in solution with trypsin in 50 mM ammonium bicarbonate. Reactions were quenched by the addition of 50% acetonitrile/5% formic acid and dried. Peptides were analysed on a Q-Exactive Plus mass spectrometer (Thermo Fisher Scientific, San Jose, CA) equipped with an Easy-nLC 1000 (Thermo Fisher Scientific), as previously reported^49^. Protein quantification was performed by Intensity Based Absolute Quantification (iBAQ)^50^. Protein intensities were calculated as the sum of all identified peptide intensities using MassChroQ^51^. Protein intensities were divided by the number of theoretically observable peptides (calculated by *in silico* protein digestion, all fully-tryptic peptides between 6 and 30 amino acids were counted while missed cleavages were neglected). Protein abundances were log2-transformed and normalized based on Htt abundance **(**Fig. S1 and Table S1).

### Bioinformatic Analysis

#### Unbinned analyses

Based on the processed protein abundance data, we applied a small sample size *t*-test by using the limma package in R 3.2.2. Then, to correct for multiple hypothesis testing, we calculated the false discovery rate (FDR) for each protein. Fold change for each protein was calculated as the ratio of average protein abundances in the expanded polyQ Htt (i.e., either Htt-97Q or Htt1-103Q) versus the Htt-25Q control pull-downs. The relationship between fold change and FDR was visualized using a log-log volcano plot. Subsequently, these two parameters (FDR and fold change) were also used to classify the proteins into three non-overlapping primary categories for binned analyses, as described below.

#### Protein classification

Proteins that were co-immunoprecipitated solely by expanded polyQ Htt (i.e., undetectable in the Htt-25Q control pull-down data set) and had FDR < 0.2, were classified as “expanded polyQ specific.” Proteins with >4-fold change (between the expanded polyQ Htt and Htt-25Q control pull-downs) and had FDR < 0.2 were classified as “expanded polyQ enriched.” Proteins with <1.5-fold change (between the expanded polyQ Htt and Htt-25Q control pull-downs) and FDR > 0.2 were classified as “expanded polyQ non-enriched.” In addition to the three primary categories, we also defined a derivative category (“expanded polyQ associated”) as the combination of the expanded polyQ specific and expanded polyQ enriched bins.

#### Binned category analyses

In order to perform global comparisons, a Fisher’s exact test was used to test the relative prevalence of the characteristic being analysed (as determined by using publicly accessible data sets listed below) among proteins of each defined category (expanded polyQ specific, expanded polyQ enriched, expanded polyQ associated and the expanded polyQ non-enriched) compared to the prevalence of the characteristic in either the whole yeast proteome or all pulled-down proteins. For comparisons between groups, a Fisher’s exact test was used to test the relative prevalence of each characteristic among the proteins in three categories (expanded polyQ specific, expanded polyQ enriched and expanded polyQ associated) compared to genes in the expanded polyQ non-enriched (control) category.

### Data Sets

All the datasets used for analyses can be found in Table 1. Where needed, we mapped the protein ID from the mass spectrometry data set to gene ID using the mapping file downloaded from EnsemblFungi (http://fungi.ensembl.org/index.html). GO-derived terms were defined by sorting all the cellular compartment-related GO terms according to broad cellular localization terms.

**Table 1:** Datasets used for Biostatistical Analyses.

Analyses performed	Dataset
Coiled-coil motif analysis	European Bioinformatics Institute website (http://www.ebi.ac.uk/reference_proteomes)
Zinc finger motif analysis	SCOP database (http://scop.mrc-lmb.cam.ac.uk/scop/)
Prion-like domain analysis	^86^
PolyQ analysis	Uniprot website (http://www.uniprot.org).
Essentiality analysis	Yeast Deletion Project (http://www-sequence.stanford.edu/group/yeast_deletion_project)
Gene Ontology analysis	Gene ontology website (http://geneontology.org/)
Protein domain annotation analysis	European Bioinformatics Institute website (http://pfam.xfam.org)
Htt toxicity analysis	^38,40–42^.
Disordered proteins analysis	DisProt (http://www.disprot.org)

### Significance Statement

This work represents the first comprehensive and unbiased analysis of protein-protein interactions of polyQ expansion proteins in cells that are susceptible to polyQ toxicity, compared to cells that are resistant to polyQ toxicity. Our results suggest that endogenous polyQ and Q/N-rich proteins play an important role in mediating cellular toxicity through protein-protein interactions, and also reveal the preferential sequestration of nucleolar and mitochondrial proteins in cells that are susceptible to polyQ toxicity.

## Results

### PolyQ expansion increases Htt interactions in both yeast species

Proteins with long polyQ stretches, such as Htt, often form aggregates. Therefore, we used a magnetic bead-capture and SDS denaturation method to identify all proteins that are preferentially bound to expanded polyQ Htt molecules, whether or not they are physically incorporated into aggregates

**Figure 1:**
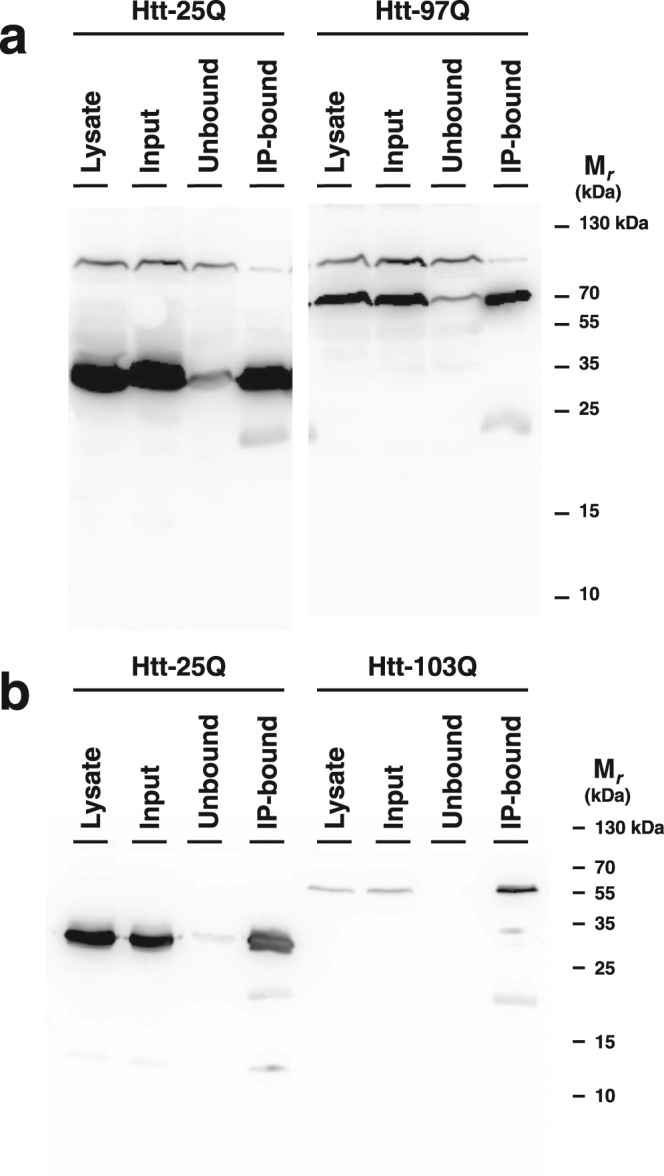
Western blot of anti-FLAG immunoprecipitation. Htt was immunoprecipitated from (**a**) *S. cerevisiae* and (**b**) *S. pombe* Htt-expression strains using an anti-FLAG mAb coupled to magnetic beads, as described in Methods. Equivalent quantities (25 OD) of whole cell lysate, input, unbound, and IP-bound samples were loaded and detected with anti-FLAG mAb. Note that the two sides of panel A are cropped from different regions of the same gel. The complete gel is shown at the end of the Supplementary Information document.

We used previously described yeast strains expressing a FLAG-tagged Htt exon 1-GFP construct with either a short (25Q) or expanded (97Q in *S. cerevisiae* or 103Q in *S. pombe*) polyQ stretch^46^. The expanded polyQ Htt proteins form aggregates as determined by microscopy in both species^46^. We employed a coupled anti-FLAG monoclonal antibody to capture Htt protein complexes from yeast cell lysates. Western blots confirmed quantitative recovery of Htt from both *S. cerevisiae* (Fig. 1a) and *S. pombe* (Fig. 1b). Of note, we observed a full recovery of Htt-97Q in *S. cerevisiae* and Htt-103Q in *S. pombe* (Fig. 1a,b, compare input vs. immunoprecipitation (IP)-bound lanes), indicating that the FLAG epitope remains accessible in expanded polyQ aggregates of Htt. Analysis of the IP-bound fraction from *S. cerevisiae* that expressed Htt-97Q (using a Coomassie-stained SDS-PAGE gel) shows enrichment of a subset of Htt-interacting proteins that differs from crude cell lysate (Fig. S2), indicating that the fraction of proteins that are able to be analysed by gel electrophoresis are distinct in our experimental samples.

**Figure 2:**
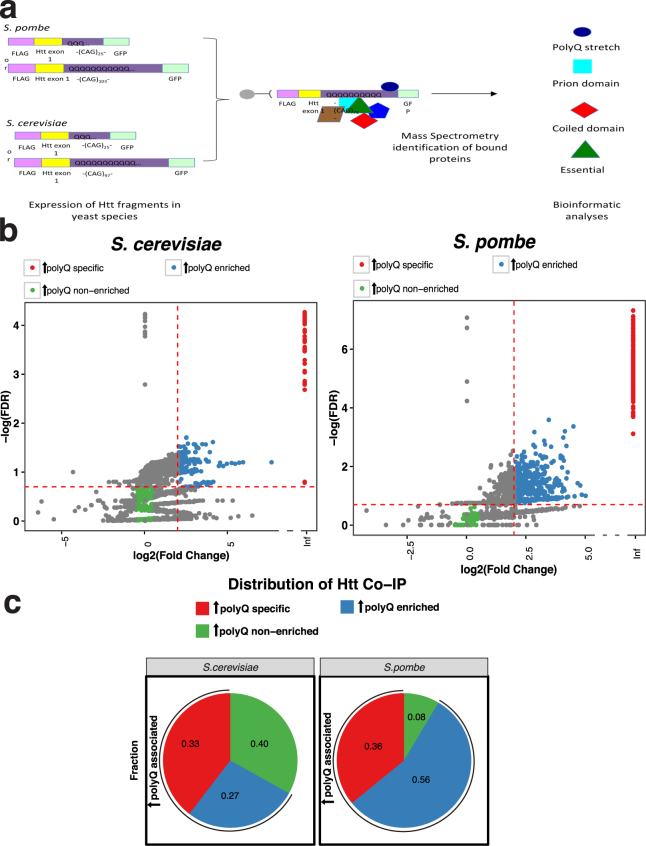
(**a**) Experimental design of proteomic analysis of Htt Co-IP proteins from *S. cerevisiae* and *S. pombe*. Inducible Htt exon 1 flanked by an N-terminal FLAG tag and a repeat stretch of 25Q or 103Q in *S. pombe* (top) or 25Q or 97Q in *S. cerevisiae* (bottom), and by a C-terminal GFP tag, was genomically integrated. Following induced expression, cultures were grown to mid-log phase and prepared for co-IP using magnetic beads with a conjugated anti-FLAG antibody. The Htt-bound specific proteins were identified and their abundance determined using a quantitative mass spectrometry approach; the data was then subjected to statistical analysis and classification. (**b**) Volcano plot of expanded co-IP proteins from *S. cerevisiae* and *S. pombe*. The volcano plots show the relation between the FDR and the fold change of expanded (designated by ↑) polyQ specific, enriched, and non-enriched groups in *S. cerevisiae* (left) and *S. pombe* (right). The Y-axis indicates the negative log10-transformed FDR of proteins in each group, and the X-axis shows log2-transformed fold change of proteins in each group. The dashed line intersecting the X-axis shows the fold change threshold to define each group and the dashed line intersecting the Y-axis shows the FDR threshold to define each group. (**c**) Protein classification of the expanded Htt co-IP proteins. Percentages of each group (expanded polyQ specific, expanded polyQ enriched, and expanded polyQ non-enriched) in the co-IP proteins from *S. cerevisiae* (left) and *S. pombe* (right).

We then used mass spectrometry (MS) to identify and quantitate endogenous proteins bound to either expanded polyQ Htt or Htt-25Q control bait in each of the IP-bound samples from both yeast species in biological triplicates (Fig. 2a and Supplementary Table S1). From these mass spectroscopy (MS) data sets, we used a rigorous combination of both fold change and FDR to characterize the proteins by comparing the relative abundance of each protein immunoprecipitated by expanded polyQ Htt versus Htt-25Q control. This analysis shows that many of the proteins detectable by MS in both species bound preferentially to expanded polyQ Htt, and a subset bound exclusively to expanded polyQ Htt (Fig. 2b). Based on the distribution of the fold change and FDR parameters (Fig. 2b), we defined three non-overlapping primary bin categories for subsequent analyses (Fig. 2b,c): expanded polyQ specific (red), expanded polyQ enriched (blue), and expanded polyQ non-enriched (green). We also defined the combination of the expanded polyQ specific and expanded polyQ enriched categories as “expanded polyQ associated” (Fig. 2c).

### Binding of essential proteins to expanded polyQ Htt in both species

Since polyQ expansion is expected to increase the overall number of Htt protein-protein interactions, it is reasonable to hypothesize that cell death in susceptible cell types might be caused by a particularly large increase in the number of aberrant interactions between expanded polyQ Htt and essential proteins. Binding to misfolded Htt could lead to bulk sequestration, mis-localization, or inactivation of such proteins, potentially compromising their essential functions.

**Figure 3:**
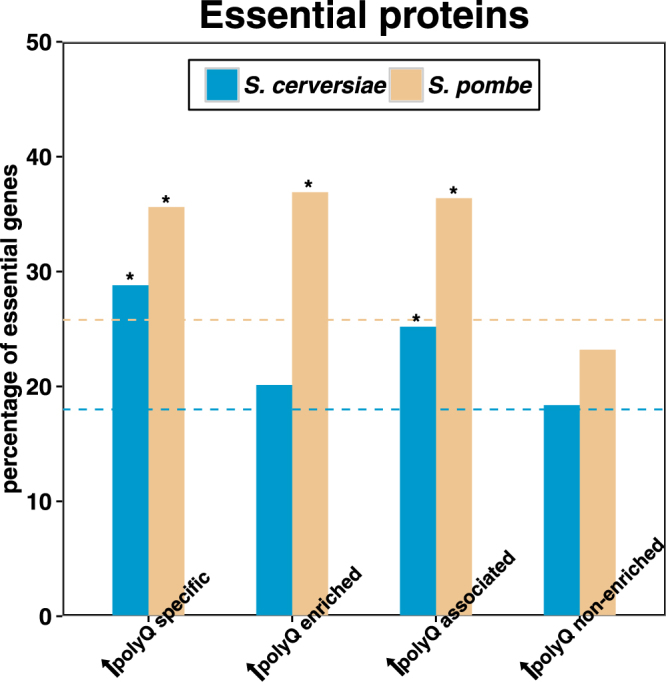
Essentiality analysis of expanded Htt co-IP proteins specific proteins. The bar-plot shows the percentage of essential genes of each group in *S. cerevisiae* (blue) and *S. pombe* (yellow). The Y-axis indicates the percentage of essential genes, and the X-axis shows the name of each group. Dashed lines show the background percentage of essential genes in the genome of *S. cerevisiae* (blue) and *S. pombe* (yellow). Asterisk shows the statistical significance in each group (*P < 0.05, **P < 0.01), calculated by the Fisher’s exact test. Detailed values and statistical results are provided in Supplementary Table 2.

We therefore analysed the effect of polyQ expansion on Htt binding to essential proteins in both *S. cerevisiae* and *S. pombe*. Our results indicated that essential proteins are overrepresented in the expanded polyQ-associated subset in both species (Fig. 3 and Supplementary Table S2). Further, whereas essential proteins comprised ~18% of all proteins in the proteome of *S. cerevisiae*, they comprised ~25% of the expanded polyQ-associated proteins in that species (representing an enrichment score of 1.40 relative to proteome and 1.08 relative to all pulled-down proteins). Similarly, whereas ~26% of all proteins in the *S. pombe* were essential, ~36% of *S. pombe* expanded polyQ-associated proteins were essential (representing an enrichment score of 1.41 relative to proteome and 1.46 relative to all pulled-down proteins).

To examine whether the observed overrepresentation of essential proteins among proteins associated with expanded polyQ Htt in both species might be due in part to preferential detection of abundant proteins by MS, we repeated our analyses using expanded polyQ non-enriched proteins as a control group for comparison. We observed no overrepresentation of essential proteins in this group in *S. cerevisiae* (enrichment score = 1.02), and a slight underrepresentation in *S. pombe* (enrichment score = 0.90) (Fig. 3 and Supplementary Table S2). Taken together, the data indicate that although essential proteins may be overrepresented in the expanded polyQ-associated subsets, the level of overrepresentation is similar between the two species of yeast. Therefore, the relative resistance of *S. pombe* to expanded Htt toxicity cannot be explained by differential binding to essential proteins.

### Localization and biological functions of proteins bound to expanded polyQ Htt differ between *S. cerevisiae* and *S. pombe*

Since a similar proportion of total essential proteins appear to associate with expanded polyQ Htt in *S. cerevisiae* and *S. pombe*, we hypothesized that the differential toxicity resulting from expanded polyQ Htt in these two species might be due to specific differences in the cellular localization and/or biological processes of the bound proteins. To examine this possibility, we used the gene ontology (GO) database to categorize and compare the localization and biological processes of proteins in the expanded polyQ-associated subsets from both species.

**Figure 4:**
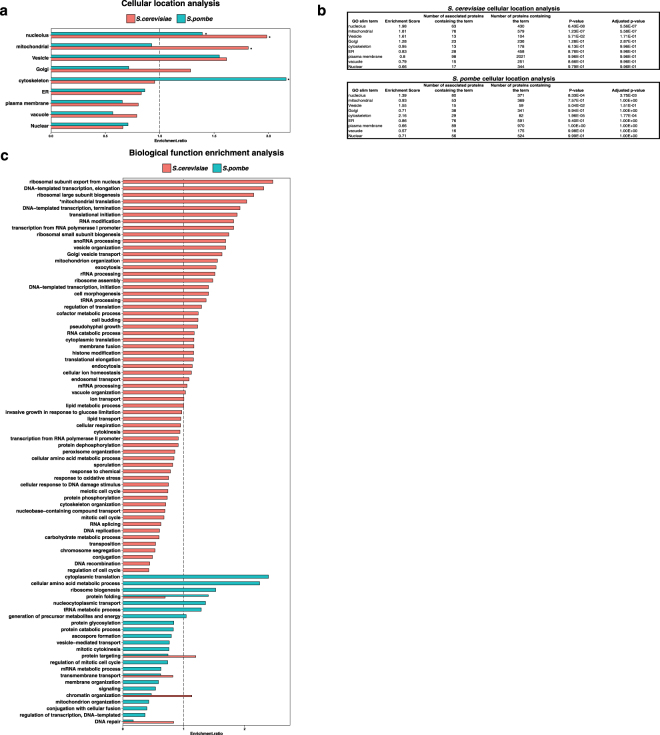
GO analysis of expanded co-IP proteins from *S. cerevisiae* and *S. pombe*. (**a**) Cellular compartment GO-derived terms (see Methods) were used to classify the cellular localization of expanded polyQ-associated group co-IP identifications; *S. cerevisiae* (red) and *S. pombe* (teal). Htt-97Q and Htt-103Q expanded polyQ-associated group co-IP identifications. The statistical significance indicated by *P < 0.05, calculated by Fisher’s exact test. (**b**) Cellular compartmentalization analysis of GO-derived terms used to classify the cellular localization of *S. cerevisiae* (top) and *S. pombe* (bottom). Statistical significance is relative to the expanded polyQ non-enriched group for each species. (**c**) GO Slim biological process enrichment analysis of Htt-97Q and Htt-103Q expanded polyQ-associated group co-IP identifications in *S. cerevisiae* (red) and *S. pombe* (teal). GO Slim terms related to biological function that overlapped between the two species were chosen for the analysis. The Y-axis shows the gene ontology terms in *S. cerevisiae* and *S. pombe*. The X-axis shows the enrichment ratio for each gene ontology term in *S. cerevisiae* and *S. pombe*. The dashed line indicates an enrichment ratio equals to 1. The statistical significance was indicated by *P < 0.05; calculated by the Fisher’s exact test.

Interestingly, we observed several marked differences in the cellular compartmentalization of expanded polyQ-associated proteins between the two species of yeast (Fig. 4a,b). Most notably, in *S. cerevisiae*, nucleolar and mitochondrial proteins were overrepresented by ~2 fold and ~1.8 fold, respectively, in the expanded polyQ-associated subset. In contrast, in *S. pombe*, nucleolar proteins were only overrepresented by ~1.4 fold in the expanded polyQ-associated subset, while mitochondrial proteins were slightly underrepresented. Cytoskeletal proteins, on the other hand, were overrepresented ~2.2 fold in the expanded polyQ-associated subset of *S. pombe*, but not significantly overrepresented in the expanded polyQ-associated subset of *S. cerevisiae*. Proteins localized to intracellular vesicles were overrepresented ~1.6-fold in the expanded polyQ-associated subsets of both species.

The overrepresentation of nucleolar and mitochondrial proteins in the expanded polyQ-associated subsets of *S. cerevisiae* was accompanied by a similar overrepresentation of biological processes that are carried out within those organelles (Fig. 4c, Supplementary Table S3). For the nucleolus, these included 5 categories from among the 10 most highly-overrepresented biological processes: ribosomal subunit export, ribosomal large subunit biogenesis, transcription from RNA polymerase I promoter, ribosomal small subunit biogenesis, and snoRNA processing. For mitochondria, these included 2 categories out of the 13 most highly-overrepresented biological processes: mitochondrial translation and mitochondrial organization. In contrast, only one biological process related to the nucleolus was overrepresented among expanded polyQ-associated proteins in *S. pombe*, and no biological processes related to mitochondria were overrepresented. The most overrepresented biological processes in *S. pombe* were cytoplasmic translation and cellular amino acid metabolism (both with enrichment ratios ~2.5) (Fig. 4c and Supplementary Table S3), and neither of these processes was overrepresented in *S. cerevisiae*.

### Binding of endogenous polyQ and prion domain proteins to expanded polyQ Htt in *S. cerevisiae*

A major difference between the proteomes of *S. cerevisiae* and *S. pombe* that might contribute to their differential susceptibility to expanded polyQ Htt-related toxicity is that *S. pombe* has very few endogenous proteins with either polyQ or prion (Q/N-rich) domains^1^. In support of this idea, previous studies have shown that several other endogenous prion-like proteins in *S. cerevisiae* appear to facilitate Htt-97Q-induced toxicity through protein-protein interactions^19,24,41^. Therefore, we performed a comprehensive and unbiased analysis of endogenous polyQ and prion-like proteins that preferentially bind expanded polyQ Htt in *S. cerevisiae*.

**Figure 5:**
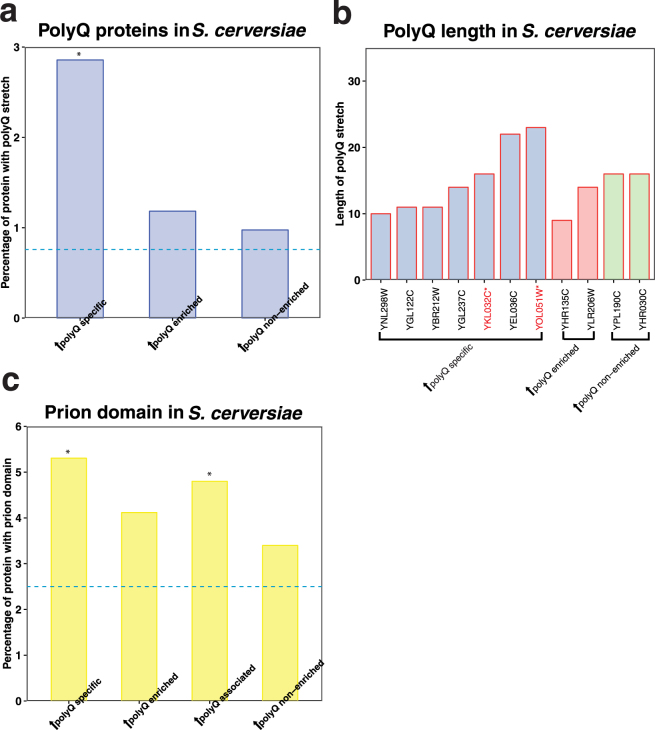
Prion domain enrichment analysis and polyQ length analysis in expanded Htt co-IP proteins. (**a**) The bar-plot shows the percentage proteins with polyQ stretch of each group in *S. cerevisiae* (no proteins with polyQ stretches were found in the *S. pombe* groups). The Y-axis indicates the percentage of proteins with polyQ stretch, and the X-axis shows the name of each group. The dashed lines show the background percentage of proteins with polyQ stretch in the proteome of *S. cerevisiae*. Asterisk shows the statistical significance in each group (*P < 0.05), calculated by the Fisher’s exact test. Detailed values and statistical results are provided in Supplementary Table 4. (**b**) The bar-plot shows the proteins with polyQ stretch of each group in *S. cerevisiae*. Y-axis indicates the length of polyQ for each protein, and X-axis shows the name of the proteins. Different groups are indicated in different colours [expanded polyQ specific (blue), expanded polyQ enriched (red), and expanded polyQ non-enriched (green) in *S. cerevisiae*]. Proteins with multiple polyQ stretches are labelled in red. (**c**) The bar-plot shows the percentage of proteins with prion domains of each group in *S. cerevisiae*. The Y-axis indicates the percentage of proteins with coiled-coil motifs, and the X-axis shows the name of each group. The dashed lines show the background percentage of proteins with prion domains in the proteome of *S. cerevisiae* (blue). Statistical significance in each group demonstrated by *P < 0.05 and **P < 0.01, calculated by the Fisher’s exact test. Detailed values and statistical results are provided in Supplementary Table 6.

Our results revealed a ~3.8-fold overrepresentation of endogenous polyQ proteins in the expanded polyQ-specific subset, compared to the whole proteome (Fig. 5a and Supplementary Table S4). Surprisingly, there did not appear to be any correlation between either the overall length or number of polyQ stretches within each protein and specific binding to Htt-97Q (Fig. 5b and Supplementary Table S5).

The results also showed a ~1.9-fold overrepresentation of endogenous prion-like proteins associated with Htt-97Q (Fig. 5c). Furthermore, the overrepresentation of both polyQ and prion domains in the expanded polyQ-associated subset of *S. cerevisiae* was also statistically significant when compared to their representation in the control expanded polyQ non-enriched subset (Supplementary Table S6). In contrast, proteins with intrinsically disordered domains were not enriched in the expanded polyQ-associated subset (Supplementary Table S7). It was not possible to perform adequately powered statistical analyses of polyQ and prion domains among expanded polyQ-associated proteins in *S. pombe* due to the natural scarcity of endogenous polyQ and prion-like proteins in that species (Supplementary Tables 5 and 6). Overall, these data confirmed that polyQ expansion causes Htt to interact with many endogenous polyQ and prion-like proteins in *S. cerevisiae*.

### The coiled-coil motif is overrepresented among expanded polyQ-associated proteins in *S. cerevisiae*

**Figure 6:**
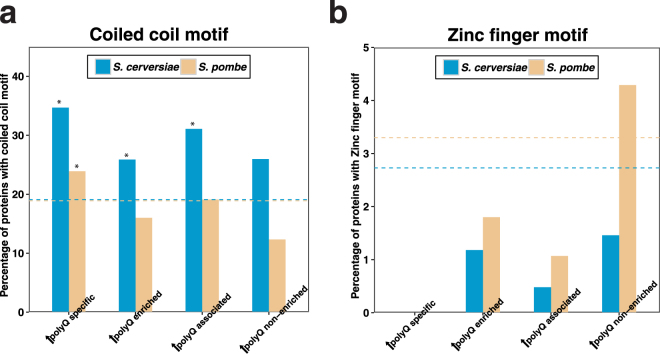
Coiled-coil motif enrichment analysis of expanded Htt co-IP proteins specific proteins. (**a**) The bar-plot shows the percentage of expanded Htt Co-IP proteins with coiled-coil motifs of each group in *S. cerevisiae* (blue) and *S. pombe* (yellow). Y-axis indicates the percentage of proteins with coiled-coil motifs in each group, and X-axis shows the name of each group. Dashed lines show the background percentage of proteins with coiled-coil motifs of each group in the proteome of *S. cerevisiae* (blue) and *S. pombe* (yellow). Statistical significance in each group indicated by *P < 0.05 and **P < 0.01, calculated using a Fisher’s exact test. Detailed values and statistical results are provided in Supplementary Table 8. (**b**) A similar enrichment test was done for zinc finger motifs for expanded Htt co-IP proteins in *S. cerevisiae* (blue) and *S. pombe* (yellow).

It has been proposed that polyQ stretches can extend coiled-coils to promote protein-protein interactions^1,4,52^. Since our data indicated that Htt-97Q preferentially binds endogenous polyQ proteins in *S. cerevisiae*, we performed an additional analysis to determine the prevalence of coiled-coil motifs among expanded polyQ-associated proteins. The results of this analysis confirmed overrepresentation of this motif in *S. cerevisiae* (~1.6 fold), but not in *S. pombe* (Fig. 6a and Supplementary Table S8). For comparison, we also analysed the relative prevalence of a different structural motif, the zinc finger, among expanded polyQ-associated proteins (Fig. 6b). In contrast to coiled-coils, the zinc finger motif was not overrepresented in expanded polyQ-associated proteins, suggesting that the interaction between coiled-coil proteins and expanded polyQ Htt is relatively specific.

### Several Htt-97Q toxicity suppressors preferentially bind expanded polyQ Htt in *S. cerevisiae*

Previous studies have identified specific genes that function as suppressors of Htt-97Q toxicity in *S. cerevisiae*^38,40–42^. We hypothesized that Htt-97Q might specifically bind to and sequester some of the proteins encoded by these suppressor genes. Therefore, we compared the relative abundance of each of these proteins within our data subsets, and identified six expanded polyQ-specific and three expanded polyQ-enriched proteins among known suppressors (Supplementary Table S9). Interestingly, a mitochondrial protein (TIM10) and a chromatin-remodelling protein (NHPB6) were among the expanded polyQ-specific proteins previously identified as functional suppressors of Htt-97Q toxicity.

## Discussion

### A comparative, proteome-wide approach to studying polyQ expansion-dependent protein-protein interactions

A leading hypothesis for the cellular toxicity of expanded polyQ proteins (like pathogenic Htt) is that they perturb endogenous protein-protein interactions^26,53–57^. In this study, we used an unbiased, systematic, and quantitative approach to compare the protein-protein interactions of expanded polyQ Htt in two yeast species that display different susceptibilities to Htt-induced toxicity^46^. Overall, a similar percentage of all essential proteins preferentially bound to expanded polyQ in both species, indicating that the difference in expanded polyQ Htt toxicity displayed by *S. cerevisiae* versus *S. pombe* cannot be explained simply by the degree to which essential proteins are sequestered. We then interrogated our data sets in greater detail to determine what more specific factors might be responsible for expanded polyQ-Htt-induced toxicity in *S. cerevisiae*. Of note, our analyses seek to identify interactions with different species of polyQ Htt, including soluble monomeric or oligomeric and aggregated species. This is important because the role of polyQ aggregation in polyQ toxicity remains unclear^16^, and we thus did not want to limit our analyses to aggregated polyQ Htt.

### Nucleolar and mitochondrial proteins preferentially bind expanded polyQ Htt in *S. cerevisiae*

We hypothesized that the observed difference in toxicity between the two yeast species might be due to specific differences in the cellular locations and functions of the bound proteins. Using gene ontology (GO) analyses, we found a striking overrepresentation of mitochondrial localization and function among expanded polyQ-associated proteins in *S. cerevisiae*, but not *S. pombe*. Although Htt aggregates are not known to enter mitochondria, they have been shown to block the import of mitochondrial proteins from the cytoplasm^58^. Therefore, expanded polyQ Htt aggregates have an opportunity to sequester mis-localized mitochondrial proteins within the cytoplasm. In addition, expanded polyQ Htt aggregates increase the rate of mitochondrial fragmentation^59^, which might allow some mitochondrial proteins to leak into the cytoplasm. Our results are consistent with a recent report showing enhanced sequestration of mitochondrial proteins in yeast expressing expanded polyQ Htt^60^. Previous studies have also shown direct interaction between expanded polyQ Htt with the outer mitochondrial membrane proteins contributes in yeast cells, leading to a dysfunctional cellular respiration^61^. More broadly, a number of other studies have also shown that changes in cellular respiration are associated with polyQ disorders and other neurodegenerative diseases^2,62–78^.

We also found a notable overrepresentation of nucleolar proteins among expanded polyQ-associated proteins in *S. cerevisiae*, which was less pronounced in *S. pombe*. Previously, we showed by dual-channel fluorescence microscopy that a fraction of expanded polyQ Htt aggregates localize to the nucleus of yeast cells, providing nucleolar proteins an opportunity to interact with Htt aggregates^46^. Recently, it has been reported that polyglutamine toxicity results in increased nucleolar stress^79^. One effect of nucleolar stress is to increase the stability of the pro-apoptotic protein p53, thus promoting cell death. While this finding would fit logically with the observed increased cell toxicity, further validation and exploration of mechanistic details are required.

In contrast, the most overrepresented cellular functions for expanded polyQ-associated proteins in *S. pombe* included cytoplasmic translation and cellular amino acid metabolic processes. Whether these interactions help to protect against Htt-103Q-mediated toxicity in *S. pombe* or are not sufficiently harmful to cause cellular toxicity remains to be investigated.

### Expanded polyQ Htt preferentially binds to endogenous polyQ and prion domain proteins

The prevalence of endogenous polyQ and prion domain (Q/N-rich) proteins is strikingly different between the proteomes of *S. cerevisiae* and *S. pombe*. Whereas >80 *S. cerevisiae* proteins possessed a polyQ stretch, there were only 3 polyQ proteins in *S. pombe*; a similarly large discrepancy in the prevalence of prion domain proteins existed between these two species.

Previous studies have suggested that proteins with pathogenic expansion of polyQ stretches can co-aggregate with normally benign endogenous polyQ proteins^1,2,24^. In our study, we found a significant overrepresentation of polyQ proteins associated with expanded polyQ Htt in *S. cerevisiae*. Somewhat surprisingly, the degree of association does not appear to correlate with the length of the polyQ stretch on the endogenous protein, suggesting that a long polyQ stretch may only be required on one of the binding partners of a heterologous polyQ-polyQ complex. Interestingly, none of the 3 endogenous polyQ proteins in *S. pombe* (Mug69, Med15, and Sol1) appeared to interact with Htt-103Q (Supplementary Table S1). In contrast, YOL051W, the *S. cerevisiae* homolog of Med15 and containing a 23Q stretch, appeared to specifically associate with Htt-97Q (Fig. 5b and Supplementary Table S5). The *S. cerevisiae* homologs of Mug69 and Sol1 are not polyQ proteins.

In addition to an overrepresentation of polyQ proteins, we also found an increased representation of prion domain-containing proteins that were selectively pulled-down by Htt-97Q in *S. cerevisiae*. Interestingly, Q/N-rich proteins are thought to be involved in the pathogenesis of a wide variety of neurodegenerative diseases caused by protein aggregation, including Alzheimer’s disease, ALS, and frontotemporal lobar degeneration with ubiquitin-only immunoreactive neuronal changes (FTLD-U). In polyglutamine diseases, prion-like proteins such as FUS/TLS have been found to aggregate in neurons^80,81^. One limitation of our approach is that it does not allow us to distinguish between direct and indirect binding partners. Therefore, we cannot determine if the observed overrepresentation of polyQ or prion-like proteins is due to specific binding of these proteins directly to the expanded polyQ stretch of Htt, or rather co-aggregation with other components of larger aggregates. Additional experiments are required to characterize these interactions in greater detail.

Our findings also revealed that coiled-coil motifs are overrepresented among expanded polyQ-associated proteins in *S. cerevisiae* but not *S. pombe*. This difference might be caused by the ability of polyQ stretches (which are nearly absent in *S. pombe*) to promote protein-protein interactions of adjacent coiled-coil domains^1,4,52^. While we were able to analyse the prevalence of coiled-coil and zinc finger motifs in our data sets, the analysis of other structural motifs is generally limited by the small size of data sets currently available for other super-secondary structural domains.

### Several genetic suppressors of Htt-97Q toxicity bind specifically to expanded polyQ Htt

A key advantage of unbiased approaches, such as the one we present, is their ability to explore multiple processes simultaneously. On the other hand, pull-down approaches are only able to identify proteins that physically interact with each other, therefore providing no guarantee of functional significance.

Other investigators have previously studied the toxicity of expanded polyQ Htt in *S. cerevisiae* by conducting screens for genes that suppress its toxicity^38,40–42^. It is possible the proteins encoded by these genes are sequestered into aggregates of expanded polyQ Htt in *S. cerevisiae*, and, therefore, overexpression of the genes would be expected restore protein levels and function. We confirmed that nine of the expanded polyQ-associated proteins in our data set were encoded by previously identified suppressor genes. One of the polyQ-associated proteins is this subgroup is TIM10, a mitochondrial protein identified in a suppressor screen by Mason *et al*.^42^. The confirmation of TIM10 as an expanded polyQ-specific protein supports the idea that mitochondrial proteins may play a central role in mediating polyQ toxicity^82–84^. Additionally, we found that NHP6B, a chromatin-remodelling protein previously identified by Giorgini *et al*. in their suppressor screen^38^ is an expanded polyQ-specific protein. This finding suggests that regulatory changes in the epigenetic control of gene expression could also play an important role in the toxicity of Htt in yeast. Interestingly, chromatin remodelling proteins are hypothesized to play a role in modulating gene expression in Huntington’s disease^85^, where they are thought to modulate neuronal gene transcription. While it is not possible to extrapolate our findings in yeast directly to the expanded polyQ toxicity seen in neurons, our data suggests that chromatin remodelling may be perturbed through aberrant protein-protein interactions.

## Conclusions

Overall, our results are consistent with a model in which the toxicity induced by expanded polyQ Htt in *S. cerevisiae* might be caused by preferential sequestration of essential nucleolar and mitochondrial proteins, perhaps mediated by physical interactions within a network of endogenous polyQ and prion-like proteins that are more abundant in *S. cerevisiae* than *S. pombe*. Our work also highlights the potential utility of evolutionarily divergent yeast species as model systems to study the general effects of polyQ expansion on protein-protein interactions and cellular functions.

## Electronic supplementary material

Supplemental Figures 1-2, Tables 2,4-8

Supplementary Table 1

Supplementary Table 3
