# Supplementary material for: Comparative Analysis of Mutant Huntingtin Binding Partners in Yeast Species
Source: Sci Rep. 2018 Jun 22;8:9554. doi: 10.1038/s41598-018-27900-5 (PMC6015068; doi:10.1038/s41598-018-27900-5)

Supplementary Information for

Comparative Analysis of Mutant Huntingtin Binding Partners in Yeast Species

Yanding Zhao, Ashley A. Zurawel, Nicole P. Jenkins, Martin L. Duennwald,

Chao Cheng, Arminja N. Kettenbach, and Surachai Supattapone

Supplementary Figures

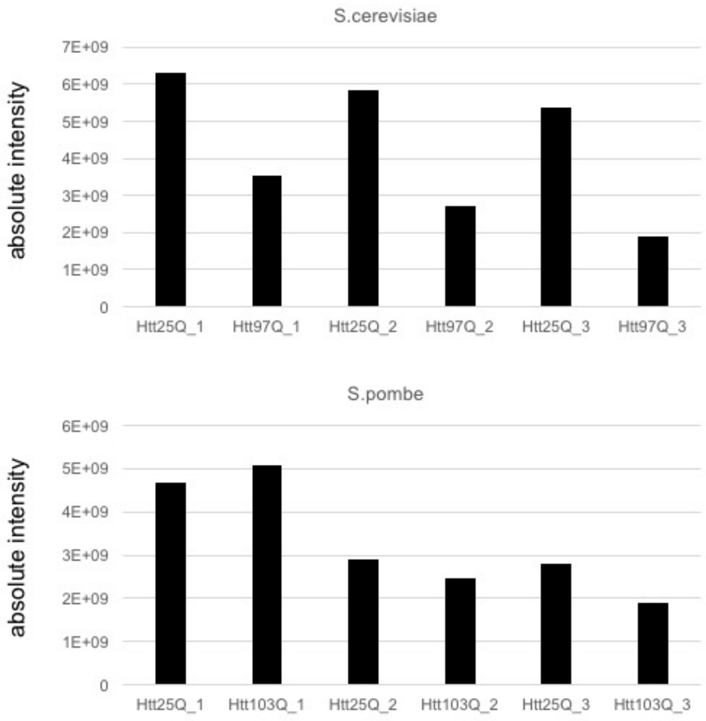

Supplementary Figure S1. Htt abundance in immunoprecipitated samples. Htt protein abundance was individually determined using iBAQ for each replicate sample (n=3) for Htt-25Q and Htt-97Q in *S. cerevisiae*, and for Htt-25Q and Htt-103Q in *S. pombe*.

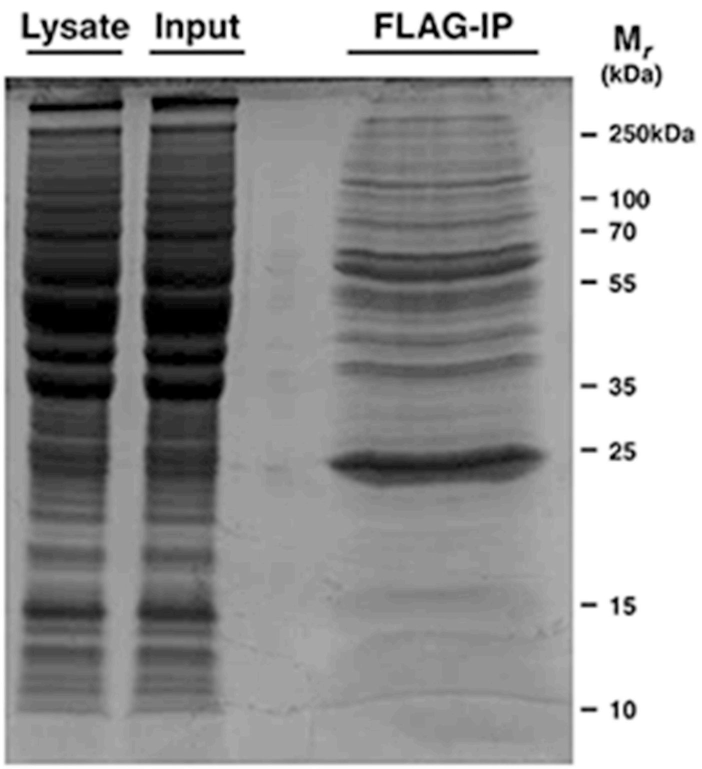

Supplementary Figure S2. Coomassie-stained SDS-PAGE of Anti-FLAG immunoprecipitation. Htt was immunoprecipitated from *S. cerevisiae* expressing Htt-97Q using an anti-FLAG mAb coupled to magnetic beads as described in Methods. Equivalent quantities (1000 OD) of whole cell lysate, input, and IP-bound samples were loaded for comparison.

Supplementary Table S2. Essentiality analysis of *S. pombe* and *S. cerevisiae* expanded Htt Co-IP proteins

Essentiality analysis of *S. pombe* expanded Htt Co-IP proteins

	Total	Essential	Non-essential	Fraction of essential genes	Enrichment Score	*P*-value
**Htt-103Q expanded polyQ specific**	292	104	188	35.60%	1.38	9E-05
**Htt-103Q expanded polyQ enriched**	440	162	278	36.82%	1.43	5E-08
**Htt-103Q associated**	732	266	466	36.33%	1.41	5E-12
**Htt-103Q expanded polyQ non-enriched**	72	16	56	22.22%	0.90	7E-01
**Proteome**	4836	1250	3586	25.80%		
**Htt-97Q expanded polyQ specific**	243	70	173	28.80%	1.23	3E-02
**Htt-97Q expanded polyQ enriched**	170	34	136	20.00%	0.86	9E-01
**Htt-97Q associated**	413	104	309	25.20%	1.08	2E-01
**Htt-97Q expanded polyQ non-enriched**	195	35	160	18.37%	0.78	1E+00
**Pull Down**	2496	585	1911	23.48%		

Essentiality analysis of *S. cerevisiae* expanded Htt Co-IP proteins

	Total	Essential	Non-essential	Fraction of essential genes	Enrichment Score	*P*-value
**Htt-97Q expanded polyQ specific**	243	70	173	28.80%	1.60	2E-05
**Htt-97Q expanded polyQ enriched**	170	34	136	20.00%	1.11	3E-01
**Htt-97Q associated**	413	104	309	25.20%	1.40	1E-06
**Htt-97Q expanded polyQ non-enriched**	195	35	160	18.37%	1.02	4E-01
**Proteome**	6174	1114	5060	18.00%		
**Htt-97Q expanded polyQ specific**	245	7	238	2.86%	1.92	6E-02
**Htt-97Q expanded polyQ enriched**	169	2	167	1.18%	0.80	7E-01
**Htt-97Q associated**	414	9	405	2.17%	1.46	1E-01
**Htt-97Q expanded polyQ non-enriched**	205	2	203	0.98%	0.66	8E-01
**Pull Down**	2252	34	2218	1.51%		

Supplementary Table S2. Essentiality analysis of *S. pombe* and *S. cerevisiae* expanded Htt co-IP proteins. Analysis was performed on expanded Htt co-IP proteins from *S. pombe* (top) and *S. cerevisiae* (bottom) using four different classifications: expanded polyQ specific, expanded polyQ enriched, associated (combination of expanded polyQ specific and expanded polyQ enriched) and expanded polyQ non-enriched. The columns indicate the number of proteins assigned to each listed group: total proteins, essential proteins, and non-essential proteins. Essentiality was defined using the Yeast Deletion Project (http://www-sequence.stanford.edu/group/yeast_deletion_project) database. The fraction of essentiality represents essential vs. total proteins (either the entire yeast proteome or all pulled-down proteins, as

indicated); the enrichment score indicates the probability of finding the essential protein in each classification compared to the chance of finding the essential proteins in the statistical background, also described by the *P*-value.

Supplementary Table S4. PolyQ stretch enrichment analysis of *S. cerevisiae* expanded Htt co-IP proteins

PolyQ stretch enrichment analysis of *S. cerevisiae* expanded Htt immunoprecipitated proteins

	Total	PolyQ	Non PolyQ	Percentage of proteins with polyQ stretch	Enrichment Score	*P*-value
**Htt-97Q expanded polyQ specific**	245	7	238	2.86%	3.77	2E-03
**Htt-97Q expanded polyQ enriched**	169	2	167	1.18%	1.56	4E-01
**Htt-97Q associated**	414	9	405	2.17%	2.86	4E-03
**Htt-97Q expanded polyQ non-enriched**	205	2	203	0.98%	1.29	5E-01
**Proteome**	6721	51	6670	0.76%		
**Htt-97Q expanded polyQ specific**	245	7	238	2.86%	1.92	6E-02
**Htt-97Q expanded polyQ enriched**	169	2	167	1.18%	0.80	7E-01
**Htt-97Q associated**	414	9	405	2.17%	1.46	1E-01
**Htt-97Q expanded polyQ non-enriched**	205	2	203	0.98%	0.66	8E-01
**Pull Down**	2252	34	2218	1.51%		

Supplementary Table S4. PolyQ stretch enrichment analysis of *S. cerevisiae* expanded Htt co-IP proteins. Analysis was performed on expanded Htt co-IP proteins from *S. cerevisiae*, using four different classifications: expanded polyQ specific, expanded polyQ enriched, associated (combination of expanded polyQ specific and expanded polyQ enriched) and expanded polyQ non-enriched. The columns indicate the number of proteins assigned to each listed group: total proteins, proteins with polyQ stretch, and proteins without. Proteins with a polyQ stretch were defined using the Uniprot (http://www.uniprot.org) database. The fraction of proteins with polyQ stretch vs. total proteins (either the entire yeast proteome or all pulled-down proteins, as indicated); enrichment score indicates the probability of finding the proteins with

at least one polyQ stretch in each classification, compared to the chance of finding the proteins with at least one polyQ stretch in the statistical background, also described by the *P*-value.

Supplementary Table S5. PolyQ length analysis of *S. cerevisiae* expanded Htt co-IP proteins

PolyQ length analysis of *S. cerevisiae* expanded Htt Co-IP proteins

Gene ID	Gene Name	Classification of Co-IP Protein	Length of PolyQ stretch
YNL298W	Cla4p	Htt-97 expanded polyQ specific	10
YBR212W	Ngr1p	Htt-97 expanded polyQ specific	11
YGL122C	Nab2p	Htt-97 expanded polyQ specific	11
YGL237C	Hap2p	Htt-97 expanded polyQ specific	14
YKL032C	Ixr1p	Htt-97 expanded polyQ specific	16
YEL036C	Anp1p	Htt-97 expanded polyQ specific	22
YOL051W	Gal11p	Htt-97 expanded polyQ specific	23
YHR135C	Yck1p	Htt-97 expanded polyQ enriched	9
YLR206W	Ent2p	Htt-97 expanded polyQ enriched	14
YHR030C	Slt2p	Htt-97 expanded polyQ non-enriched	16
YPL190C	Nab3p	Htt-97 expanded polyQ non-enriched	16

Supplementary Table S5. PolyQ length analysis of *S. cerevisiae* expanded Htt co-IP proteins. Analysis was performed on expanded Htt co-IP proteins from *S. cerevisiae,* using four different classifications: expanded polyQ specific, expanded polyQ enriched, associated and expanded polyQ non-enriched (as previously defined). The columns indicate the gene name encoding the proteins containing more than 10 consecutively repeated glutamine residues, its function, and its group classification. The length of polyQ stretch shows the number of repeated glutamines in the encoded proteins from the co-IP.

Supplementary Table S6. Prion domain analysis of *S. pombe* and *S. cerevisiae* expanded Htt co-IP proteins

Prion domain enrichment analysis of *S. pombe* expanded Htt co-IP proteins

	Total	Prion	Non Prion	Percentage of proteins with prion domains	Enrichment Score	*P*-value
**Htt-103Q expanded polyQ specific**	293	2	291	0.07%	1.6	4E-01
**Htt-103Q expanded polyQ enriched**	450	0	450	0.00%	0	1E-00
**Htt-103Q associated**	743	2	741	0.03%	0.63	9E-01
**Htt-103Q expanded polyQ non-enriched**	73	0	73	0.00%	0	1E+00
**Proteome**	5145	22	5123	0.43%		
**Htt-103Q expanded polyQ specific**	293	2	291	0.70%	1.71	3E-01
**Htt-103Q expanded polyQ enriched**	450	0	450	0.00%	0	1E-01
**Htt-103Q associated**	741	2	0	0.30%	0.67	9E-01
**Htt-103Q expanded polyQ non-enriched**	73	0	73	0.00%	0	1E+00
**Pull Down**	2252	9	2243	0.40%		

Prion domain enrichment analysis of *S. cerevisiae* expanded Htt co-IP proteins

	Total	Prion	Non Prion	Percentage of proteins with prion domains	Enrichment Score	*P*-value
**Htt-97Q expanded polyQ specific**	245	13	232	5.30%	2.13	8E-03
**Htt-97Q expanded polyQ enriched**	170	7	163	4.10%	1.65	1E-01
**Htt-97Q associated**	415	20	395	4.80%	1.93	3E-03

**Htt-97Q expanded polyQ non-enriched**	206	7	199	3.39%	1.36	3E-01
**Proteome**	6692	167	6525	2.50%		
**Htt-97Q expanded polyQ specific**	245	13	232	5.30%	1.45	1E-01
**Htt-97Q expanded polyQ enriched**	170	7	163	4.10%	1.13	4E-01
**Htt-97Q associated**	415	20	395	4.80%	1.32	1E-01
**Htt-97Q expanded polyQ non-enriched**	206	7	199	3.50%	0.93	9E-01
**Pull Down**	2545	93	2452	3.70%		

Supplementary Table S6. Prion domain analysis of *S. pombe* and *S. cerevisiae* expanded Htt Co-IP proteins. Analysis was performed on expanded Htt co-IP proteins from *S. pombe* (top) and *S. cerevisiae* (bottom) using four different classifications: expanded polyQ specific, expanded polyQ enriched, associated and expanded polyQ non-enriched, as previously defined. The columns indicate the number of proteins assigned to each listed group: total proteins, proteins with prion domain, and unknown proteins. The proteins containing prion domains were defined as by using the method described by Harrison and Gerstein [1]. The fraction of prion domains represents proteins with coiled-coil domains vs. total proteins (either the entire yeast proteome or all pulled-down proteins, as indicated); enrichment score indicates the probability of finding the proteins with a prion domain in each classification, compared to the chance of finding the proteins with a prion domain in the statistical background, also described by the *P*-value.

Supplementary Table S7. Disordered protein analysis of *S. pombe* and *S. cerevisiae* expanded Htt co-IP proteins

Disordered protein analysis of *S. pombe* expanded Htt co-IP proteins

	Total	Disordered	Non-Disordered	Fraction of disordered proteins	Enrichment Score	*P*-value
**Htt-103Q expanded polyQ specific**	293	0	293	0.00%	0	1
**Htt-103Q expanded polyQ enriched**	450	1	449	0.20%	11.43	0.09
**Htt-103Q associated**	743	1	742	0.10%	6.92	0.14

**Htt-103Q expanded polyQ non-enriched**	73	0	73	0.00%	0	1
**Proteome**	5145	1	5144	0.00%		
**Htt-103Q expanded polyQ specific**	293	0	293	0.00%	0	1
**Htt-103Q expanded polyQ enriched**	450	1	449	0.20%	5.00	0.2
**Htt-103Q associated**	743	1	742	0.10%	3.03	0.33
**Htt-103Q expanded polyQ non-enriched**	73	0	73	0.00%	0	1
**Pull Down**	2252	1	2251	0.00%		

Disordered protein analysis of *S. cerevisiae* expanded Htt co-IP proteins

	**Total**	**Disordered**	**Non-Disordered**	**Fraction of disordered proteins**	**Enrichment Score**	***P*-value**
**Htt-97Q expanded polyQ specific**	245	2	243	0.82%	1.56	0.4
**Htt-97Q expanded polyQ enriched**	170	0	170	0.0%	0.0	1.0
**Htt-97Q associated**	415	2	413	0.48%	0.92	0.65
**Htt-97Q expanded polyQ non-enriched**	206	2	204	0.97%	1.86	0.3
**Proteome**	6704	35	6669	0.52%		
**Htt-97Q expanded polyQ specific**	245	2	243	0.82%	0.9	0.7
**Htt-97Q expanded polyQ enriched**	170	0	170	0.0%	0.0	1

**Htt-97Q associated**	415	2	413	0.48%	0.53	0.9
**Htt-97Q expanded polyQ non-enriched**	206	2	204	0.97%	1.07	0.6
**Pull Down**	2545	23	2522	0.90%		

Supplementary Table S7. Disordered protein analysis of *S. pombe* and *S. cerevisiae* expanded Htt co-IP proteins. Analysis was performed on expanded Htt co-IP proteins from *S. pombe* (top) and *S. cerevisiae* (bottom) using four different classifications: expanded polyQ specific, expanded polyQ enriched, associated (combination of expanded polyQ specific and expanded polyQ enriched) and expanded polyQ non-enriched. The columns indicate the number of proteins assigned to each listed group: total proteins, disordered proteins and non-disordered proteins. Disordered proteins were defined using the DisProt (http://www.disprot.org) database. The fraction of disordered proteins represents disordered proteins vs. total proteins (either the entire yeast proteome or all pulled-down proteins, as indicated); the enrichment score indicates the probability of finding the disordered protein in each classification compared to the chance of finding the disordered proteins in the statistical background, also described by the *P*-value.

Supplementary Table S8. Coiled-coil motif analysis of *S. pombe* and *S. cerevisiae* expanded Htt co-IP proteins

Coiled-coil motif enrichment analysis of *S. pombe* expanded Htt co-IP proteins

	Total	Coiled-coil	Non coiled-coil	Percentage of proteins with coiled-coil domains	Enrichment Score	*P*-value
**Htt-103Q expanded polyQ specific**	293	70	223	23.90%	1.26	2E-02
**Htt-103Q expanded polyQ enriched**	450	72	378	16.00%	0.85	1E+00
**Htt-103Q associated**	743	142	601	19.10%	1.01	5E-01
**Htt-103Q expanded polyQ non-enriched**	73	9	64	12.30%	0.65	1E+00
**Proteome**	5145	972	4173	18.90%		

**Htt-103Q expanded polyQ specific**	293	70	223	23.90%	1.12	2E-01
**Htt-103Q expanded polyQ enriched**	450	72	378	16.00%	0.75	1E+00
**Htt-103Q associated**	743	142	601	19.10%	0.89	1E+00
**Htt-103Q expanded polyQ non-enriched**	73	9	6*4	12.30%	0.58	1E+00
**Pull Down**	2252	481	1771	21.40%		

Coiled-coil domain enrichment analysis of *S. cerevisiae* expanded Htt co-IP proteins

	**Total**	**Coiled-coil**	**Non coiled-coil**	**Percentage of proteins with coiled-coil domains**	**Enrichment Score**	***P*-value**
**Htt-97Q expanded polyQ specific**	245	85	160	34.70%	1.82	3E-09
**Htt-97Q expanded polyQ enriched**	170	44	126	25.60%	1.35	1E-02
**Htt-97Q associated**	415	129	160	31.08%	1.62	1E-09
**Htt-97Q expanded polyQ non-enriched**	208	54	154	26.34%	1.39	8E-03
**Proteome**	6692	1279	5213	19.10%		
**Htt-97Q expanded polyQ specific**	245	85	160	34.70%	1.40	1E-04
**Htt-97Q expanded polyQ enriched**	170	44	126	25.60%	1.05	4E-01
**Htt-97Q associated**	415	129	160	31.08%	1.26	7E-04

**Htt-97Q expanded polyQ non-enriched**	208	54	154	26.34%	1.05	4E-01
**Pull Down**	2549	630	1919	24.70%		

Supplementary Table S8. Coiled-coil domain analysis of *S. pombe* and *S. cerevisiae* expanded Htt co-IP proteins. Analysis was performed on expanded Htt co-IP proteins from *S. pombe* (top) and *S. cerevisiae* (bottom) using four different classifications: expanded polyQ specific, expanded polyQ enriched, associated and expanded polyQ non-enriched, as previously defined. The columns indicate the number of proteins assigned to each listed group: total proteins, proteins with coiled-coil domains and unknown proteins. Proteins with coiled-coil domains were found using the http://www.ebi.ac.uk/reference proteome database. The fraction of coiled-coil domains represents proteins with one/more coiled-coil domain vs. total proteins (either the entire yeast proteome or all pulled-down proteins, as indicated); the enrichment score indicates the probability of finding the proteins with a coiled-coil domain in each classification, compared to the chance of finding the proteins with a coiled-coil domain in the statistical background, also described by the *P*-value.

Supplementary Table S9. Toxicity analysis of *S. cerevisiae* expanded Htt co-IP proteins

Genes in *S. cerevisiae* that suppress HTT-97Q mediated toxicity

	Gene Symbol	Mol Weight	Length	HTT-25Q Average Abundance	HTT-97Q Average Abundance	Fold Change	Protein classification
Giorgini *et* al.	NHP6B	11386.98	99	NA	22.63	NA	Htt-97Q expanded polyQ specific
Kayatekin et al.	UBP3	100124.18	912	NA	18.63	NA	Htt-97Q expanded polyQ specific
Mason *et* al.	RRP46	24117.88	223	NA	21.07	NA	Htt-97Q expanded polyQ specific
	TIM10	10151.88	93	NA	20.34	NA	Htt-97Q expanded polyQ specific
	GCS1	38758.16	352	18.66	21.86	3.20	Htt-97Q expanded polyQ enriched
	MGDP1	20266.45	178	19.82	22.21	2.39	Htt-97Q expanded

							polyQ enriched
	TIM9	10096.98	87	22.06	22.03	-0.03	Htt-97Q expanded polyQ non-enriched
	GAL7	41860.50	366	27.52	27.16	-0.36	Htt-97Q expanded polyQ non-enriched
	SEE1	28349.48	257	18.80	19.03	0.23	Htt-97Q expanded polyQ non-enriched
Wolfe et al.	MIG1	54216.20	504	NA	19.83	NA	Htt-97Q expanded polyQ specific
	YGP1	36471.39	354	21.57	24.79	3.22	Htt-97Q expanded polyQ enriched
	NAB3	89293.54	802	19.75	19.89	0.15	Htt-97Q expanded polyQ non-enriched

Supplementary Table S9. Toxicity analysis of *S. cerevisiae* expanded HTT co-IP proteins.
Expanded Htt co-IP proteins were analysed for finding toxicity-related proteins as reported by Giorgini *et al.* [2], Kayatekin *et al.* [3], Mason *et al.* [4], and Wolfe *et al.* [5]. The resulting proteins are listed by their Gene ID for *S. cerevisiae*. Protein classification for expanded Htt toxicity-related proteins is listed on the left. Note that protein abundance levels are log2 transformed. Therefore, the fold change is calculated by taking the difference of between log2-transformed average abundance levels.

References

1 Harrison, P. M. & Gerstein, M. A method to assess compositional bias in biological sequences and its application to prion-like glutamine/asparagine-rich domains in eukaryotic proteomes. *Genome Biol* **4**, R40, doi:10.1186/gb-2003-4-6-r40 (2003).
2 Giorgini, F., Guidetti, P., Nguyen, Q., Bennett, S. C. & Muchowski, P. J. A genomic screen in yeast implicates kynurenine 3-monooxygenase as a therapeutic target for Huntington disease. *Nature genetics* **37**, 526-531, doi:ng1542 [pii]
10.1038/ng1542 [doi] (2005).
3 Kayatekin, C. *et al.* Prion-like proteins sequester and suppress the toxicity of huntingtin exon 1. *Proc Natl Acad Sci U S A* **111**, 12085-12090, doi:10.1073/pnas.1412504111 [doi]
1412504111 [pii] (2014).
4 Mason, R. P. *et al.* Glutathione peroxidase activity is neuroprotective in models of Huntington's disease. *Nature genetics* **45**, 1249-1254, doi:10.1038/ng.2732 [doi]
ng.2732 [pii] (2013).
5 Wolfe, K. J., Ren, H. Y., Trepte, P. & Cyr, D. M. Polyglutamine-rich suppressors of huntingtin toxicity act upstream of Hsp70 and Sti1 in spatial quality control of amyloid-like proteins. *PLoS One* **9**, e95914, doi:10.1371/journal.pone.0095914 (2014).

Complete Blot for Figure 1A

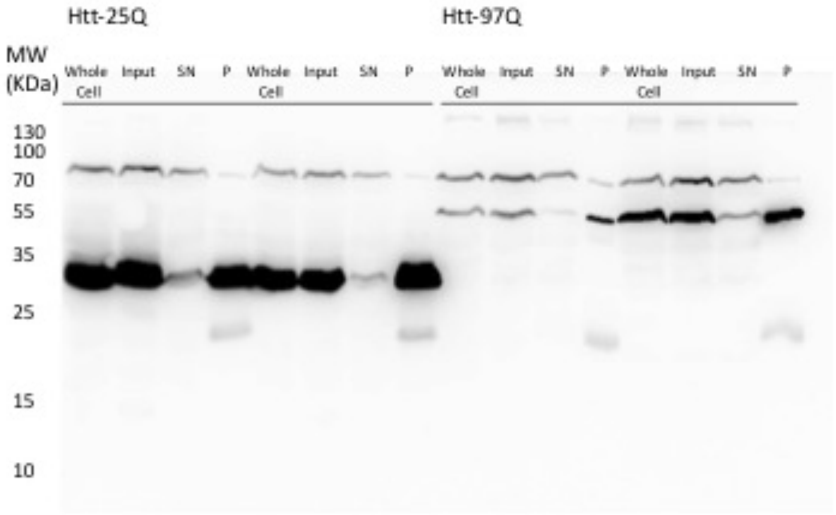

Supplement: Supplementary file 1 — Supplemental Figures 1-2, Tables 2,4-8 [file 41598_2018_27900_MOESM1_ESM.pdf]
